# Homozygous missense *WIPI2* variants cause a congenital disorder of autophagy with neurodevelopmental impairments of variable clinical severity and disease course

**DOI:** 10.1093/braincomms/fcab183

**Published:** 2021-09-03

**Authors:** Reza Maroofian, Andrea Gubas, Rauan Kaiyrzhanov, Marcello Scala, Khalid Hundallah, Mariasavina Severino, Mohamed S Abdel-Hamid, Jill A Rosenfeld, Darius Ebrahimi-Fakhari, Zahir Ali, Fazal Rahim, Henry Houlden, Sharon A Tooze, Norah S Alsaleh, Maha S Zaki

**Affiliations:** Department of Neuromuscular Diseases, University College London, Queen Square, Institute of Neurology, London, UK; Goethe University Medical School, University Hospital, 60590 Frankfurt am Main, Germany; Department of Neuromuscular Diseases, University College London, Queen Square, Institute of Neurology, London, UK; Department of Neuromuscular Diseases, University College London, Queen Square, Institute of Neurology, London, UK; Department of Neurosciences, Rehabilitation, Ophthalmology, Genetics, Maternal and Child Health, University of Genoa, Genoa, Italy; Pediatric Neurology and Muscular Diseases Unit, IRCCS Istituto Giannina Gaslini, Via Gerolamo Gaslini, Genoa, Italy; Division of Neurology, Department of Pediatrics, Prince Sultan Military Medical City, Riyadh, Saudi Arabia; Neuroradiology Unit, IRCCS Istituto Giannina Gaslini, Genoa, Italy; Medical Molecular Genetics Department, Human Genetics and Genome Research Division, National Research Centre, Cairo, Egypt; Department of Molecular and Human Genetics, Baylor College of Medicine, Houston, TX, USA; Baylor Genetics Laboratories, Houston, TX, USA; Department of Neurology, The F.M. Kirby Neurobiology Center, Boston Children’s Hospital, Harvard Medical School, Boston, MA 02115, USA; Laboratory for Genome Engineering, Division of Biological Sciences, 4700 King Abdullah University of Science and Technology, Thuwal 23955-6900, Saudi Arabia; Department of Physiology, Bacha Khan Medical College, Mardan, Pakistan; Department of Neuromuscular Diseases, University College London, Queen Square, Institute of Neurology, London, UK; The Francis Crick Institute, Molecular Cell Biology of Autophagy, NW1 1AT London, UK; Division of Medical Genetics and Metabolic Medicine, Department of Pediatrics, Prince Sultan Military Medical City, 11159 Riyadh, Saudi Arabia; Human Genetics and Genome Research Division, Clinical Genetics Department, National Research Centre, 12311 Cairo, Egypt

**Keywords:** *WIPI2*, WIPI2b, autophagy, neurodevelopmental disorder, congenital disorders of autophagy

## Abstract

*WIPI2* is a member of the human WIPI protein family (seven-bladed b-propeller proteins binding phosphatidylinositols, PROPPINs), which play a pivotal role in autophagy and has been implicated in the pathogenesis of several neurological conditions. The homozygous *WIPI2* variant c.745G>A; p.(Val249Met) (NM_015610.4) has recently been associated with a neurodevelopmental disorder in a single family. Using exome sequencing and Sanger segregation analysis, here, two novel homozygous *WIPI2* variants [c.551T>G; p.(Val184Gly) and c.724C>T; p.(Arg242Trp) (NM_015610.4)] were identified in four individuals of two consanguineous families. Additionally, follow-up clinical data were sought from the previously reported family. Three non-ambulant affected siblings of the first family harbouring the p.(Val184Gly) missense variant presented with microcephaly, profound global developmental delay/intellectual disability, refractory infantile/childhood-onset epilepsy, progressive tetraplegia with joint contractures and dyskinesia. In contrast, the proband of the second family carrying the p.(Arg242Trp) missense variant, similar to the initially reported *WIPI2* cases, presented with a milder phenotype, encompassing moderate intellectual disability, speech and visual impairment, autistic features, and an ataxic gait. Brain MR imaging in five patients showed prominent white matter involvement with a global reduction in volume, posterior corpus callosum hypoplasia, abnormal dentate nuclei and hypoplasia of the inferior cerebellar vermis. To investigate the functional impact of these novel *WIPI2* variants, we overexpressed both in *WIPI2*-knockout HEK293A cells. In comparison to wildtype, expression of the Val166Gly WIPI2b mutant resulted in a deficient rescue of LC3 lipidation whereas Arg224Trp mutant increased LC3 lipidation, in line with the previously reported Val231Met variant. These findings support a dysregulation of the early steps of the autophagy pathway. Collectively, our findings provide evidence that biallelic *WIPI2* variants cause a neurodevelopmental disorder of variable severity and disease course. Our report expands the clinical spectrum and establishes *WIPI2-*related disorder as a congenital disorders of autophagy.

## Teaser

Maroofian et al. reported two novel homozygous *WIPI2* variants [c.551T>G; p.(Val184Gly) and c.724C>T; p.(Arg242Trp) (NM_015610.4)] in four individuals of two consanguineous families with neurodevelopmental disorder and brain abnormalities. Expression of the Val184Gly mutant in *WIPI2*-knockout HEK293A cells resulted in a deficient LC3 lipidation rescue whereas Arg242Trp mutant increased LC3 lipidation.

## Graphical Abstract

**Graphical Abstract:**
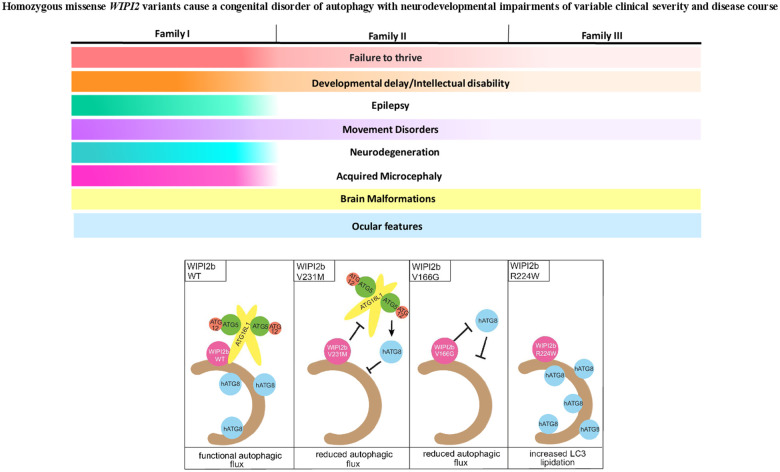

## Introduction

The human WIPI family encompasses 4 members (WIPI1-4) known as seven-bladed β-propeller proteins that bind phosphatidylinositols (PROPPINs). These proteins bind phosphatidylinositol-3-phosphate (PI(3)P) and phosphatidylinositol-3,5-bisphosphate (PI(3,5)P2) and are involved in autophagy.^1–3^ Pathogenic variants in the *WIPI* genes are associated with several neurological conditions (Supplementary Table 1). *De novo* variants in *WIPI4* (*WDR45*, OMIM #300526) cause an X-linked neurodegenerative condition known as beta*-*propeller protein*-*associated neurodegeneration (BPAN, OMIM #300894).^4^^,^^5^ Biallelic variants in *WIPI3* (*WDR45B*, OMIM #609226) cause a syndrome known as a neurodevelopmental disorder with spastic quadriplegia and brain abnormalities with or without seizures (NEDSBAS, OMIM #617977), characterized by progressive neurological deterioration with pyramidal and extrapyramidal features as well as musculoskeletal abnormalities.^6^ Additionally, *de novo* loss-of-function missense variants in *WIPI1* (OMIM #609224) have recently been detected in a large cohort of anencephalic cases, suggesting a possible role in embryonic brain development and neural tube formation.^7^ Very recently, both monoallelic and biallelic variants in *WIPI2* (OMIM #609225) have been reported to be associated with neurological conditions.^8^^,^^9^

WIPI2 (mainly the WIPI2b isoform) plays a crucial role in the formation of the phagophore, the initial step of the highly-conserved, self-degradative, and dynamic recycling process, known as autophagy.^10^^,^^11^ WIPI2b isoform is 18 amino acids shorter than the main, full-length isoform, WIPI2a, where Val184Gly and Arg242Trp mutations correspond to Val166Gly and Arg224Trp, respectively. According to studies on the yeast orthologue HSV2, WIPI2 exhibits a PI(3)P- and (PI(3,5)P2)-binding activity within the FRRG motif, at 2 sites, (site 1 and site 2), localized on blades 5 and 6.^9^^,^^12^^,^^13^ After the PI(3)P-rich omegasome is generated from the endoplasmic reticulum, WIPI2 (WIPI2b and WIPI2d isoforms) recruits the ATG12–ATG5–ATG16L1 complex to the phagophore, enabling LC3 lipidation and fostering autophagosome formation.^1^^,^^14^ Proper functioning of the autophagic molecular machinery is essential for neuronal homeostasis and survival.^11^ Abnormal autophagy is involved in the pathogenesis of several neurodegenerative disorders and single-gene defects in key autophagy proteins lead to childhood-onset neurological disorders.^15–17^

The *de novo* missense variant c.914A>G; p.(Tyr305Cys) (NM_015610.4) in *WIPI2* was first reported as a candidate for cerebral palsy after its identification in a female with hemiplegia, hydrocephalus and periventricular leukomalacia.^8^ Subsequently, the homozygous missense variant c.745G>A; p.(Val249Met) (NM_015610.4) was identified as the cause of a new neurodevelopmental disorder in four individuals from a large consanguineous family living in a remote area of Northern Pakistan.^9^ Clinical data in this family were scarce and limited to only two affected individuals (Table 1). The disorder is currently known as Intellectual Developmental Disorder with Short stature and variable Skeletal Anomalies (IDDSSA, OMIM #618453), characterized by impaired intellectual development, behavioural abnormalities, ventriculomegaly, dysmorphic features, mild skeletal abnormalities.^9^

**Table 1:** Summary of genetic and clinical features of cases with *WIPI2*-related NDD

Families (Ancestry)	Family I (Egypt)			Family II (Saudi Arabia)	Jelani et al.^9^; **1 Family (Pakistan)**	
Individuals	**F1-IV**:**1**	**F1-IV**:**3**	**F1-IV**:**4**	**F2-IV:-3**	**NA (IV-4)**	**AA (V-3)**
Age*, sex*	13 years, F (deceased at 14 y)	11.4 years, M	7.5 years, F	5 years, F	50 years, M	47 years, M
Consanguinity	+	+	+	+	+	+
*WIPI2 variant*	c.551T>G;	c.551T>G;	c.551T>G;	c.724C>T;	c.745G>A;	c.745G>A;
*(*NM_015610.4)	p.(Val184Gly)	p.(Val184Gly)	p.(Val184Gly)	p.(Arg242Trp)	p.(Val249Met)	p.(Val249Met)
*Status*	Hom	Hom	Hom	Hom	Hom	Hom
Pregnancy	Uneventful	Uneventful	Uneventful	Preterm birth (30 weeks)	Uneventful	Uneventful
Growth parameters at birth	Normal	Normal	Normal	Normal	Normal weight reported	Normal weight reported
Growth parameters at last evaluation	W 12.5 kg (−3.5 SDs)	W 11 kg (−3.1 SDs)	W 11 kg (−3 SDs)	W 16.8 kg (−0.17 SDs)	N/A	N/A
	H 110 cm (−7 SDs)	H 106 cm (−5.8 SDs)	H 101 cm (−4 SDs)	H 101.5 cm (−0.84 SDs)	N/A	N/A
	OFC 47 cm (−4.9 SDs)	OFC 46.5 cm (−4.9 SDs)	OFC 47.5 cm (−3.1 SDs)	OFC 48.5 cm (−0.71 SDs)	N/A	N/A
Congenital MC	−	−	−	−	N/A	N/A
Acquired MC	+	+	+	−	N/A	N/A
Feeding difficulties	+	+	+	−	N/A	N/A
Development						
Motor	Severely delayed	Severely delayed	Severely delayed	Moderatly delayed	Delayed	Delayed
Speech	Severely delayed	Severely delayed	Severely delayed	Severely delayed	Delayed	Delayed
Social	Severely delayed	Severely delayed	Severely delayed	Severely delayed	Delayed	Delayed
Regression	−	+ (at 1.5 y)	−	−	−	−
ID	+, profound	+, profound	+, profound	+, severe	+, moderate	+, moderate
Behavioural disturbances	Excessive crying, irritability, poor sleep	Excessive crying, irritability, poor sleep	Irritability	Stereotyped behaviour, irritability, excessive crying, self-injurious behaviour	Enuresis, nocturia, abnormal rational thinking, ISB	Enuresis, nocturia, abnormal rational thinking, ISB
Seizures				−	−	−
Onset	2 years	3 years	Infancy			
Type	GMS, TS	GMS, TS	MS			
Frequency	Weekly	Weekly	Occasional			
Refractory	+	+	N/A			
Abnormal EEG	+	+	+	−	−	−
Hypotonia	−	−	−	−	−	−
Spastic tetraplegia	+	+	+	−	−	−
Hyperreflexia	+	+	+	−	−	−
Movement disorders	Dyskinesia	Dyskinesia	Dyskinesia	Ataxic gait	Dysarthria and mild gait ataxia	Dysarthria and mild gait ataxia
Other neurological problems	Rigidity, drooling	Rigidity, drooling	Rigidity	Rigidity	Impaired memory	Impaired memory
Facial dysmorphism	+	+	+	−	+	+
Short stature	+	+	+	−	Mild	Mild
Musculoskeletal abnormalities	+	+	+	−	Mild	Mild
Muscle wasting	+	+	+	−	−	−
Ocular features	Nystagmus	Nystagmus	Nystagmus	Nystagmus, suspected CRD	Nystagmus, cataracts, age-related macular degeneration	Nystagmus, cataracts, age-related macular degeneration
Abnormal ECG	+	+	+	−	+	+
Endocrinological features	−	−	−	−	Subclinical hypothyroidism	Subclinical hypothyroidism
Other clinical features	Hemolytic anaemia, dysphagia and feeding difficulties	Joint contructures, dysphagia and feeding difficulties	Joint contructures, dysphagia and feeding difficulties	Constipation	Mild digit abnormalities, Variable subclinical cardiac arrythmias	Mild digit abnormalities, Variable subclinical cardiac arrythmias
Neuroimaging						
− WM volume loss	+	+	+	+	+	+
− Enlarged ventricles	+	+	+	+	+	+
− CCH	+	+	+	+	+	N/A
− WM signal alterations	+	+	−		N/A	N/A
− IVH	+	+	+	+	+	N/A
− CDN abnormalities	+	+	+	−	−	N/A
− Platyspondyly	+	+	+	+	−	N/A

CCH, corpus callosum hypoplasia; CDN, cerebellar dentate nuclei; CRD, cone–rod dystrophy; ECG, electrocardiogram; EEG, electroencephalogram; F, female; GMS, generalized myoclonic seizures; H, height; Hom, homozygous; ID, intellectual disability; ISB, inappropriate sexual behaviour; IVH, inferior vermis hypoplasia; M, male; MC, microcephaly; MS, myoclonic seizures; N/A, not available; OFC, occipito-frontal circumference; SDs, standard deviations; TS, tonic seizures; W, weight; WM, white matter.

Here, we report four individuals from two consanguineous families harbouring two ultra-rare homozygous *WIPI2* missense variants and presenting with a neurodevelopmental disorder. In addition, we re-evaluate data from previously reported cases with a homozygous *WIPI2* variant including additional clinical details and brain MRI findings. Using functional assays in cultured cells, we demonstrated that disease-associated *WIPI2* variants cause dysregulation of autophagy. Collectively, these findings confirm the causality of biallelic missense variants in *WIPI2* and expand the molecular and phenotypic spectrum of this emerging congenital disorder of autophagy.

## Materials and methods

### Clinical and genetic studies

**Figure 1:**
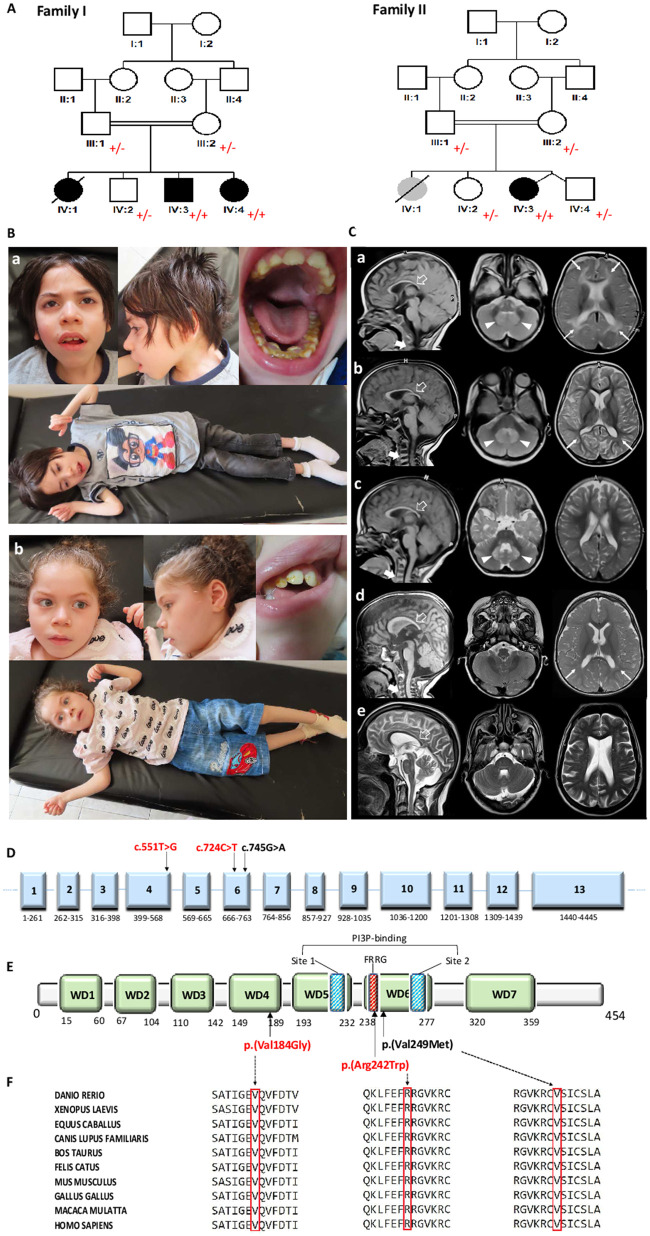
**Molecular, clinical and neuroimaging findings in patients with homozygous variants in WIPI2.** (**A**) Pedigree showing consanguinity within the two families and the genotypes of tested individuals, indicated as + (mutated) and − (wild type). (**B**) Clinical images of the siblings from Family 1, F1-IV:3 (a), and F1-IV:4 (b). Case F1-IV:3 has microcephaly, a severe spastic tetraplegia, contractures of the hands/wrist and ankles, and diffuse muscle wasting. Dysmorphic features include a long face with a prominent chin, thick eyebrows, prominent nose, thick alveolar ridge, dental deformities and large ears. Case F1-IV:4 is microcephalic and presents with severe spastic tetraplegia, distal upper and lower extremity contractures, and muscle atrophy. Her dysmorphic features are milder and mainly consisting of a long face, prominent nose, long philtrum, retrognathia and large ears with prominent antihelix. (**C**) Brain MRIs of the reported subjects from Family 1: F1-IV:1 (a), F1-IV:3 (b), and F1-IV:4 (c); Family 2: F2-IV:3 (d); and AA (V-3) (e) from Jelani et al.^9^ In all cases, the sagittal T_1_- and T_2_-weighted scans show hypoplasia of the corpus callosum, with predominant involvement of the posterior sections (empty arrows) and small inferior cerebellar vermis. In Case F1-IV:1 (a), axial T_2_-weighted images show a reduction of the white matter bulk with mild ventriculomegaly, enlargement of the frontotemporal subarachnoid spaces and deep white matter hyperintensity (arrows). Similar findings, with less prominent white matter signal alterations, can be observed in the axial T_2_-weighted images of Cases F1-IV:3 (b), F1-IV:4 (c) and F2-IV:3 (d). Marked swelling with T_2_-hyperintensity of the cerebellar dentate nuclei is noted in Cases F1-IV:1 (a), F1-IV:3 (b) and F1-IV:4 (c) (arrowheads). In Cases F1-IV:1 (a), F1-IV:3 (b), F1-IV:4 (c) and F2-IV:3 (d), sagittal T_1_-weighted images reveal platyspondyly of the cervical vertebrae (thick arrows). (**D**) Schematic drawing of the longest *WIPI2* transcript (NM_015610.4) consisting of 4445 nucleotides in 13 exons. *WIPI2* variants are shown in black (previously reported patients) or in red (this study). (**E**) The WIPI2 protein (NP_056425.1, isoform a, WIPI2a) consists of 454 amino acids encompassing seven WD repeat domain (seven-bladed b-propeller protein). The conserved arginine residues of the FRRG sequence in the blades 5 and 6 bind two head groups on the PI(3)P participating in the two distinct pockets in blade 5 (site 1) and 6 (site 2), which play a pivotal role in the binding of phosphatidylinositols. Amino acid changes are indicated in black (previous cases) and red (this study). (**F**) Conservation of the affected amino acid residues among different species according to Polyphen-2 (http://genetics.bwh.harvard.edu/pph2/). Gene transcript and protein details are available at https://www.ensembl.org (WIPI2-201, transcript ID ENST00000288828.9), https://www.nextprot.org (NX_Q9Y4P8), https://www.uniprot.org (Q9Y4P8), https://www.proteomicsdb.org (Q9Y4P8).

In this study, we evaluated two independent consanguineous families of Egyptian and Saudi origin (Fig. 1A). We also collected follow-up data from two previously reported cases from a Pakistani family.^9^ The study was approved by the institutional ethics committees of the participating centres and written informed consent was obtained from the families, in accordance with the Declaration of Helsinki. Detailed clinical features as well as family history were obtained from all affected individuals and reviewed carefully by a group of clinical geneticists (M.Z. and N.A.) and pediatric neurologists (M.S., R.K., H.H. and D.F.E.). Brain MRIs were reviewed by an experienced neuroradiologist (M.S.). Clinical exome sequencing and Sanger segregation analysis were performed independently at two different accredited diagnostic laboratories, Centogene and Baylor Genetics.

### Cell culture and plasmids

HEK293A wildtype and *WIPI2* CRISPR KO cells (Gubas et al., unpublished data) were maintained in Dulbecco’s Modified Eagle’s Medium, supplemented with 10% Fetal Bovine Serum and 1% Penicillin and Streptomycin. The cells were transiently transfected for 24 h using Lipofectamine 2000 (Invitrogen) according to the manufacturer’s protocol. To induce amino acid starvation, Earle’s Balanced Salt Solution (EBSS) was used for two hours in the presence or absence of 100 nM Bafilomycin A1 (Calbiochem). pcDNA3.1 was used as an empty vector control. WIPI2b-HA WT plasmid was a kind gift from Prof. Adi Kimchi, Weizmann Institute, Israel, and was previously described.^18^ V166G and R224W mutants were generated by site-directed mutagenesis. Primers used—V166G (Forward 5′ cgaccatcggagaggggcaggtcttcgatac; Reverse 5′gtatcgaagacctgcccctctccgatggtcg) and R224W (Forward 5′ ccagaaggacaaaaactctttgagttttggagaggagtaaag; Reverse 5′ ctttactcctctccaaaactcaaagagtttttgtccttctgg).

### Western blot

Whole cell lysates were prepared using TNTE lysis buffer [20 mM Tris-HCl, pH 7.5, 150 mM NaCl, 5 mM EDTA and 0.3% Triton-X100, supplemented with 1× Complete protease inhibitor cocktail (Roche) and 1× PhosSTOP (Roche)]. Lysates were cleared by centrifugation and 4× SDS-Sample buffer was added to the clear supernatant. Samples were boiled for 5 min at 95°C. Proteins were loaded in equal amounts and resolved on Tris-Glycine 4–12% gels (Bio-Rad), following transfer to a PVDF membrane (Millipore). Following incubations with primary and secondary antibodies, the blots were developed using Luminol reagent (Santa Cruz). Mouse antibodies used in this study: anti-WIPI2 (2A2 clone Abcam, ab105459) and Vinculin (Sigma, V9264). Rabbit antibodies used in this study: anti-p62 (Enzo Life Sciences, PW9860) and anti-LC3B (Abcam, ab48394). Densitometry was performed with ImageJ software.

### Statistical analysis

Statistics were performed using GraphPad Prism 9 software. Statistical analysis was performed by one-way ANOVA with Tukey’s post-test, from three independent experiments.

### Data availability

The data that support the findings of this study are available from the corresponding authors upon request.

## Results

### Clinical and neuroimaging findings

Family 1 consists of three affected siblings (two females and one male) born to consanguineous parents of Egyptian origin (Table 1). Elder affected sibling (F1-IV:1) was a 13-year-old girl who first presented with severe global developmental delay and hypotonia during infancy. Starting at the age of 2 years, she has suffered from recurrent and refractory generalized myoclonic and tonic seizures. She displayed microcephaly, non-specific dysmorphic facial features and mild musculoskeletal abnormalities (kyphoscoliosis, pes planus, overlapping toes and contractures of the wrists and hands) (Supplementary Table 2). Neurological examination revealed nystagmus, drooling, dysphagia, a spastic tetraplegia with hyperreflexia, as well as dyskinesia (Fig. 1B). EEG showed an abnormal background with bilateral temporoparietal epileptiform discharges. At 10 years of age, she developed a refractory and severe haemolytic anaemia of unknown aetiology and succumbed to pneumonia-related respiratory failure 3 years later.

Case F1-IV:3 is an 11.4-year-old male sibling, who presented with mild developmental delay in infancy followed by regression at the age of 1.5 years. Since the age of 3, he has suffered from recurrent weekly myoclonic and tonic seizures despite treatment with multiple anti-epileptic drugs. Similar to his sister, his EEG showed bilateral temporal epileptiform activity. Physical examination revealed microcephaly, non-specific dysmorphic facial features, kyphoscoliosis, long fingers and toes, and distal contractures of the hands and ankles (Fig. 1B). Similar to his sibling, his neurological examination showed a non-verbal patient with a combination of severe spastic tetraplegia and intermittent dyskinesia, with significant muscle atrophy and signs of bulbar dysfunction such as drooling and dysphagia (Video 1).

The youngest sibling (F1-IV:4) is a 7.5-year-old girl with profound intellectual disability. She had myoclonic seizures since early infancy with temporoparietal epileptiform discharges and abnormal slowing background on EEG. Neurological findings were similar to her siblings (Fig. 1B and Video 2).

Family II consists of a 5-year-old female index case (F2-IV:3) born to consanguineous parents of Saudi origin (Table 1). At 11 months, she was diagnosed with global developmental delay and nystagmus. Electroretinography and visual evoked potentials showed bilateral visual pathway involvement suggestive of cone–rod dystrophy. At 5 years of age, she was able to walk with support with a broad-based ataxic gait. The speech was limited to about 20 words. She displayed stereotyped movements, self-injurious behaviours and autistic features. A formal neuropsychological assessment was not performed but her intelligence quotient was estimated to be around 50.

Two affected brothers, reported previously by Jelani et al.,^9^ were re-assessed as part of this study. No additional features or regression were noticed since the last examination about 5 years prior. At the ages of 47 and 50 years, both patients present with moderate intellectual disability, behavioural abnormalities, hypomimia, bradykinesia, a mildly ataxic gait, subtle dysmorphic features and short stature along with vision impairment caused by cataracts and age-related macular degeneration (Videos 3 and 4). Their two affected cousins, also homozygous for the p.(Val249Met) variant, passed away of an undetermined cause at the ages of 54 and 38 years.

Brain MRI studies of all 4 cases and of one of the cases reported by Jelani et al.^9^ revealed corpus callosum hypoplasia with predominant involvement of the splenium. There was a mild to moderate reduction of the periventricular white matter and enlargement of the lateral ventricles in all cases (Fig. 1C). Hypoplasia of the inferior cerebellar vermis and enlargement of frontotemporal subarachnoid spaces was also noted. Non-specific white matter signal changes were observed in 3/5 patients. Marked swelling and T_2_ hyperintensity of the cerebellar dentate nuclei were present in affected siblings from Family 1 (3/5 cases). In 4/5 patients, platyspondyly was noticed at the cervical level.

### Molecular findings

In Family 1, clinical exome sequencing for the index case was carried out at Centogene as previously described,^19^ leading to the identification of a novel homozygous missense variant, c.551T>G; p.(Val184Gly) (NM_015610.4), in *WIPI2* (Fig. 1D). Sanger Sequencing confirmed the segregation of this variant with the phenotype within the family. The variant is absent from gnomAD, NHLBI Exome Sequencing Project (ESP), and Centogene and Baylor Genetics databases, as well as in Queen Square Genomics database of ∼20 000 exomes. The variant involves a highly conserved residue (GERP score 5.78 and CADD score 25.8) (Fig. 1F) and is predicted to be damaging/deleterious by most *in-silico* tools (Supplementary Table 3). Valine at position 184 localizes to a beta-sheet within blade 4 and its substitution with glycine could impact hydrogen bond formation, leading to abnormal blade folding, membrane association, or likely disruption of protein–protein interactions (Fig. 1E).

In Family 2, clinical exome sequencing for the index case was performed at Baylor Genetics as previously described,^20^ leading to the identification of the homozygous missense variant c.724C>T; p.(Arg242Trp) (NM_015610.4) in *WIPI2* (Fig. 1D). The variant was validated by Sanger sequencing and segregated with the phenotype in all family members. This variant was absent from gnomAD, ESP, Baylor Genetics database and Queen Square Genomics database of ∼20 000 exomes. It is predicted to be damaging/deleterious by most of the employed *in-silico* tools (Supplementary Table 2). Arginine at position 242 is a highly conserved residue within the FRRG motif (GERP score 5.6 and CADD score 24.8) (Fig. 1F), known to be essential for WIPI2 binding to PIPs (Fig. 1E). The substitution of this residue with threonine has been previously reported to block WIPI2b puncta formation during autophagy, resulting in autophagy inhibition.^1^ The introduction of a hydrophobic residue, such as tryptophan, might have the opposite effect of a threonine substitution, and potentially promote PIP or membrane binding at a higher rate than fusion and degradation occur, which could lead to accumulation of cargo and aggregates.

In both families, no additional biallelic variants of likely pathogenic significance were identified.

### Functional studies of novel missense variants

To investigate the functional consequences of the identified *WIPI2* variants, we overexpressed both mutants in HEK293A cells lacking *WIPI2* (WIPI2 KO) and looked at LC3 lipidation. The cells lacking WIPI2 show a severe defect in LC3 lipidation, which is supported by previous reports.^1^ We looked if we can rescue LC3 lipidation in these cells by overexpressing WIPI2b WT and the two mutant variants.

**Figure 2:**
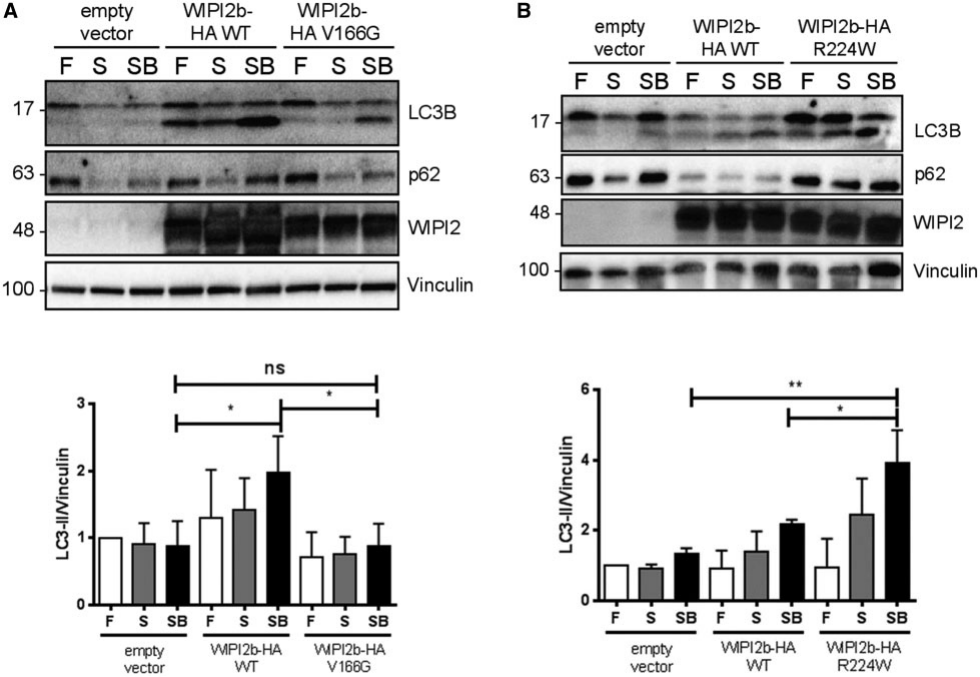
**The analysis of autophagic flux upon overexpression of WIPI2b mutants.***WIPI2* KO HEK293A cells transiently expressing empty vector, WIPI2b-HA WT or either (**A**) WIPI2b-HA V166G or (**B**) WIPI2b-HA R224W, were left untreated or treated with EBSS (amino acid starvation) or EBSS with Bafilomycin A1 for 2 h. The cells were lysed and subjected to SDS-PAGE and Western blot. Antibodies to LC3B, p62, WIPI2 and vinculin were used as indicated. *N* = 3, representative experiment is shown. Statistical analysis of LC3-II levels for each blot was performed by one-way ANOVA with Tukey’s post-test. SEM from *n* = 3. **P < 0.01. F—fed, untreated; S—EBSS starvation; SB—EBSS starvation + Bafilomycin A1.

Interestingly, when we overexpressed WIPI2b-HA wild type (WT) and WIPI2b V166G (corresponding to V184G in the longest isoform of WIPI2, WIPI2a) we observed that WIPI2b V166G mutant failed to rescue LC3 lipidation (Fig. 2A). p62 was used as a marker for autophagic cargo degradation. Expectedly, WIPI2b-HA WT was able to rescue LC3 lipidation. Further experiments are required to better define the underlying mechanism of action, and understand if this variant impedes (PI(3)P) binding or inhibits interaction with ATG16L1 or other binding partners. However, considering that the mutation is located on the blade 4, it is likely that association with another protein is affected.^21^

Overexpression of WIPI2b R224W (corresponding to R242 in WIPI2a) mutant in WIPI2 KO cells induced LC3 lipidation, which was even stronger than LC3 lipidation caused by *WIPI2* WT overexpression (Fig. 2B). We observed differences between conditions [untreated, amino acid starvation (starved) or starved with Bafilomycin A1, where Bafilomycin A1 is used as an inhibitor of the fusion between autophagosomes and lysosomes], suggesting dysregulation of autophagic flux when *WIPI2* variants are expressed, but also that some degree of autophagic flux is maintained (Fig. 2B). This is further supported by the strong accumulation of p62 cargo marker upon rescue with R224W mutant.

## Discussion

**Figure 3:**
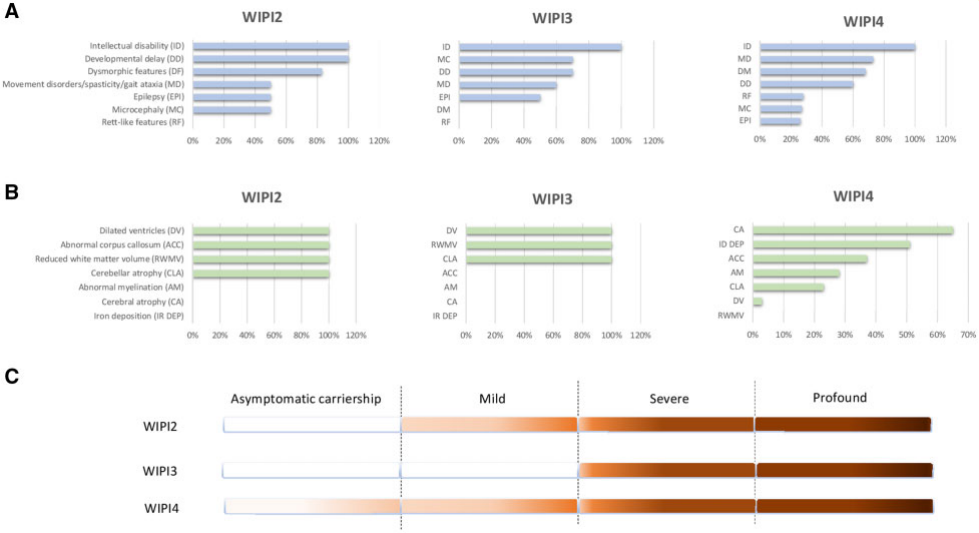
**Phenotypic comparison between WIPI2, WIPI3 and WIPI4.** (**A**) Frequency of symptoms. (**B**) Frequency of neuroradiological findings. (**C**) Range of phenotype severity. The actual numbers leading to the displayed percentages are provided in Supplementary Table 1.

Congenital disorders of autophagy are known to cause multisystem diseases in children with early and severe involvement of the CNS. Neurological manifestations are typically broad, but preferential degeneration of the cerebellar Purkinje cells and long-projecting cortical neurons is seen.^16^ Current knowledge of the clinical phenotypes associated with the defective WIPI proteins mostly comes from *WDR45* (WIPI4)-associated BPAN, which was first reported almost a decade ago.^22^^,^^23^ For phenotypic comparison associated with defects in *WIPI2*, *WIPI3 and WIPI4* genes refer to Fig. 3 and Supplementary Table 1. Cumulative phenotypic analysis of 64 individuals reported by Stige et al.^24^ and 123 individuals reported by Adang et al.^25^ (∼85% are females) with disease-causing *WIPI4 (WDR45)* variants has suggested a highly variable phenotype ranging from a severe and early disease to asymptomatic carriers, which is typically seen in females, most likely due to skewed X-inactivation. In its classic form, BPAN tends to have a biphasic course including developmental delay and seizures predominating in early childhood, followed by a progressive decline of neurological and cognitive functions frequently associated with dystonia and parkinsonism. Early childhood-onset refractory epilepsy, including myoclonic epilepsy, is relatively common. Variable features include limb spasticity, visual impairment, and rarely dysmorphic features including but not limited to microcephaly, hypertelorism, bilateral low-set ears, kyphosis, tapered fingers with fifth finger clinodactyly. Although the clinical data are currently limited to only 10 reported cases, *WIPI3* appears to also manifest as an early infantile-onset progressive disease that can be seen in the progressive microcephaly and quadriplegia associated with a progressive cortical and white matter loss on brain MR imaging.^6^ Evidence for *WIPI3*-related neurodegeneration comes from the *wipi3*-deficient mice model, which suggested a degree of neuronal loss that is more severe than in *wipi4*-deficient mice. Additionally, *wipi3*-deficient mice show prominent cerebellar damage, a phenotype that is yet to be described in cases carrying *WIPI3* variants.^26^ Thus, both *WIPI3 and WIPI4* manifest as a spectrum of early-onset neurodevelopmental disorders and later neurodegeneration.

The phenotypes of the families with *WIPI2* variants described in the present report were mainly characterized by the early CNS involvement. Although global developmental delay, intellectual disability, posterior corpus callosum hypoplasia and the inferior cerebellar vermis hypoplasia were uniform features, significant interfamilial variability in the clinical spectrum and severity of the disease is noted. Siblings from Family 1 presented with a phenotype similar to *WIPI3* cases from the Suleiman et al.^6^ report and the initial phase of the severely affected *WIPI4* cases. Apart from an early motor regression in Case F1-IV:3, suggestive of a neurodegenerative course, no significant interfamilial variability was present in Family 1. Contrasting this, affected individuals from Family 2 and report by Jelani et al.^9^ presented with a milder non-progressive phenotype including mild-to-moderate gait ataxia and retinal abnormalities as well as moderate developmental delay and intellectual disability. Despite the significant phenotypic variability and a limited number of cases, defective *WIPI2*, similar to other congenital disorders of autophagy, seems to preferentially impact cortical pyramidal neurons and the corticospinal tracts (Family 1 with severe and progressive spasticity) as well as cerebellar Purkinje cells (nystagmus and the inferior cerebellar vermis hypoplasia in all families, and ataxic gait in Case F2-IV:3 and the affected individuals reported by Jelani et al.^9^).

Neuroimaging findings were consistent with the prominent involvement of the white matter. In particular, we identified a thinning of the corpus callosum with prevalent posterior hypoplasia, variably associated with reduced white matter volume and periventricular signal alterations. Of note, long white matter tracts, including the corpus callosum and corticospinal tracts, are frequently involved in inborn disorders of autophagy.^16^ In addition, we noted a striking involvement of the cerebellar dentate nuclei that appeared swollen and hyperintense on T_2_ weighted images in the most severe cases of Family 1. Cerebellar dentate nuclei abnormalities might thus be an imaging marker of clinical severity in WIPI2 deficiency, that need to be confirmed in larger clinical series. Remarkably, *wdr45 and wdr45b* KO mice exhibit swollen axons with spheroid accumulation in the deep cerebellar nuclei.^27^ However, studies based on animal models are needed to verify if similar histological features are also present in WIPI2 deficiency. Finally, we found platyspondyly of the cervical vertebrae in the majority of our patients, further straightening the phenotypic overlap between autophagy disorders and lysosomal storage diseases.^16^ Although the exact mechanisms are still unclear, it has been demonstrated that dysregulation of the autophagic response is involved in the pathogenesis of diseases of bone (Paget disease) and cartilage (osteoarthritis and the mucopolysaccharidoses).^28^ In particular, vertebral body abnormalities, including wedge-shaped vertebral bodies, anterior beaking with posterior scalloping and platyspondyly, are observed in subjects with multiple sulfatase deficiency, a very severe form of mucopolysaccharidoses due to mutations in the *SUMF1* (sulfatase modifying factor 1) gene.^28^ Multiple measurements of autophagy in the chondrocytes of *sumf1*^−/−^ mice recapitulating the human skeletal anomalies revealed severe lysosomal vacuolization and an increased number of autophagosomes compared with wild-type chondrocytes,^29^^,^^30^ providing evidence that abnormal autophagic activity has an impact on the normal growth plate and bone growth.

Taken together, our report expands the phenotypic spectrum of WIPI2-associated disease and highlights severe manifestations shared with other disorders of autophagy, including those caused by *WIPI3 and WIPI4.*

**Figure 4:**
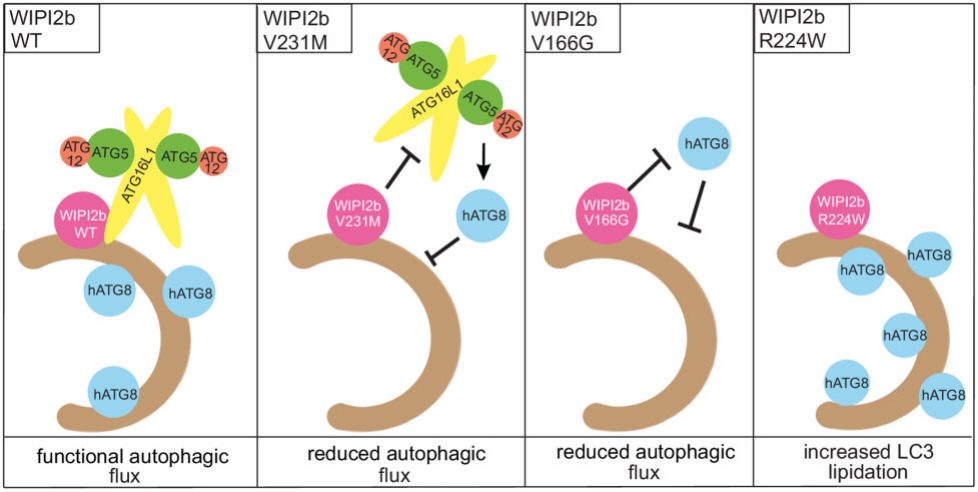
**Model summarizing the functional phenotype of the three *WIPI2* variants.** WIPI2b during autophagy associates with the phagophore through (PI(3)P) binding. WIPI2b binds ATG16L1, thereby recruiting the ATG12–ATG5–ATG16L1 complex to the phagophore in order to direct the lipidation of human ATG8 proteins, such as LC3s or GABARAPs to the correct location on the membrane containing phosphatidyethanolamine (PE). The previously reported mutation, WIPI2b Val231Met (Val249Met in WIPI2a), has been shown to inhibit the binding to ATG16L1, which leads to a reduction in the lipidation of LC3s and GABARAPs (showed by reduced formation of LC3-positive puncta in patient-derived fibroblasts) (Jelani et al.^9^), a step required for the elongation of the phagophore and subsequent formation of an autophagosome. WIPI2b Val166Gly (Val184Gly in WIPI2a) expression decreased LC3 lipidation, which reduces autophagy flux. WIPI2b Arg224Trp (Arg242Trp in WIPI2a) induced LC3 lipidation.

To explore the phenotypic differences among *WIPI2* patients (Table 1), we assessed the functional impact of the discovered variants. The p.(Val249Met) variant affects a conserved residue on the surface of the blade 6 adjacent to site 2, leading to abnormal ATG16L1 binding and membrane interaction as a result of impaired PI(3)P and PI(3,5)P2 binding.^9^ The p.(Val184Gly) variant identified in family 1 affects a conserved residue within the blade 4 and the equivalent mutant in WIPI2b is unable to rescue LC3 lipidation fully to the level of WT. Although this domain is not directly implicated in the hitherto identified autophagy-related WIPI2 functions, a loss of function would explain the severe and progressive neurological phenotype as well as the cerebellar dentate nuclei involvement observed in Family 1. The p.(Arg242Trp) variant within the FRRG motif detected in Family 2 might instead lead to dysregulation of early steps of autophagy. Future studies will have to determine the precise molecular impact of this and other missense variants. The functional phenotype of the WIPI2 missense variants reported here is summarized in Fig. 4. Our findings support a loss-of-function mechanism in *WIPI2*-related disorder, in line with the loss-of-function constraint metrics of *WIPI2* (pLOF 0.62) and similarly to other *WIPI* genes (*WIPI1*, pLOF 0.65; *WIPI3*, pLOF 0.55; *WIPI4*; pLOF 0.21).

WIPI2 phosphorylation is important for neuronal autophagosome biogenesis and is negatively affected by ageing.^31^ An age-dependent decline in neuronal autophagy is implicated in several age-dependent neurodegenerative conditions and can be rescued by *WIPI2* overexpression.^31^^,^^32^ This would suggest that individuals with *WIPI2* deficiency might be more prone to early neuronal degeneration, similarly to *WIPI3 and WIPI4*.^5^^,^^6^

In summary, our study supports that biallelic *WIPI2* variants cause a congenital disorder of autophagy, with a wide clinical spectrum and variable disease severity, possibly explained by the differential impact of different variants on autophagic initiation and flux. Larger, longitudinal studies will be needed to systematically define clinical manifestations of *WIPI2*-associated disorder. A better understanding of the disease manifestations and the underlying molecular mechanisms will enable the development of targeted therapeutic approaches in the future.

### Web resources

The following URLs were used for data presented herein:

Centogene; https://www.centogene.com/pharma/mutation-database-centomd.html

ClinVar; https://www.ncbi.nlm.nih.gov/clinvar

Combined Annotation Dependent Depletion (CADD); http://cadd.gs.washington.edu

Ensembl; https://www.ensembl.org/index.html

NHLBI GO Exome Sequencing Project (ESP); https://evs.gs.washington.edu/EVS/

Gene Cards; http://www.genecards.org

Genome Aggregation Database (GnomAD); http://gnomad.broadinstitute.org

Genomic Evolutionary Rate Profiling; http://mendel.stanford.edu/SidowLab/downloads/gerp

Greater Middle East (GME) Variome Project; http://igm.ucsd.edu/gme

Iranome; http://www.iranome.ir

Mutalyzer; https://mutalyzer.nl

Mutation Assessor; http://mutationassessor.org/r3

Mutation Taster; http://www.mutationtaster.org

NeXtProt; https://www.nextprot.org

Online Mendelian Inheritance in Man; http://www.ncbi.nlm.nih.gov/Omim

Polyphen-2; http://genetics.bwh.harvard.edu/pph2

Proteomics DB; https://www.proteomicsdb.org

PubMed; http://www.ncbi.nlm.nih.gov/pubmed

RefSeq; https://www.ncbi.nlm.nih.gov/refseq

SIFT; https://sift.bii.a-star.edu.sg

The 1000 Genomes Browser; http://browser.1000genomes.org/index.html

The Greater Middle East (GME) Variome Project; http://igm.ucsd.edu/gme/index.php

UniProt; https://www.uniprot.org

UCSC Human Genome Database; http://www.genome.ucsc.edu

Varsome; https://varsome.com

## Supplementary material

Supplementary material is available at *Brain Communications* online.

## Supplementary Material

fcab183_Supplementary_Data

## Abbreviations

ATG12 =: autophagy-related 12
ATG16L1 =: autophagy 16-like 1
ATG5 =: autophagy-related 5
BPAN =: beta-propeller protein-associated neurodegeneration
CADD =: combined annotation-dependent depletion
CRISPR =: Clustered Regularly Interspaced Short Palindromic Repeats
EBSS =: Earle's Balanced Salt Solution
EDTA =: ethylenediamine tetraacetic acid
ESP =: Exome Sequencing Project
FRRG =: Phenylalanine-Arginine-Arginine-Glycine
GERP =: Genomic Evolutionary Rate Profiling
HEK293A =: human embryonic kidney 293 A
IDDSSA =: Intellectual Developmental Disorder with Short stature and variable Skeletal Anomalies
KO =: knock-out
LC3 =: Microtubule-associated protein 1A/1B-light chain 3
LC3B =: microtubule-associated protein 1A/1B-light chain 3 B
MR =: magnetic resonance
NEDSBAS =: Neurodevelopmental disorder with spastic quadriplegia and brain abnormalities with or without seizures
NHLBI =: National Heart, Lung and Blood Institute
OMIM =: Online Mendelian Inheritance in Man
PhosSTOP =: phosphatase inhibitors
PI(3)P =: phosphatidylinositol 3-phosphate
PI(3,5)P2 =: phosphatidylinositol 3,5-bisphosphate
pLOF =: Predicted loss-of-function
PVDF =: polyvinylidene difluoride
SDS =: sodium dodecyl sulfate
SUMF1 =: Sulfatase Modifying Factor 1
WDR45 =: WD repeat-containing protein 45
WDR45B =: WD repeat-containing protein 45 B
WIPI =: WD40 repeat protein interacting with phosphoinositides
WT =: wild type
