# Supplementary material for: Homozygous missense WIPI2 variants cause a congenital disorder of autophagy with neurodevelopmental impairments of variable clinical severity and disease course
Source: Brain Commun. 2021 Sep 3;3(3):fcab183. doi: 10.1093/braincomms/fcab183 (PMC8453401; doi:10.1093/braincomms/fcab183)
Supplement: fcab183_Supplementary_Data [file fcab183_Supplementary_Data.zip › Supplementary Figure.pdf]

Supplementary Figure

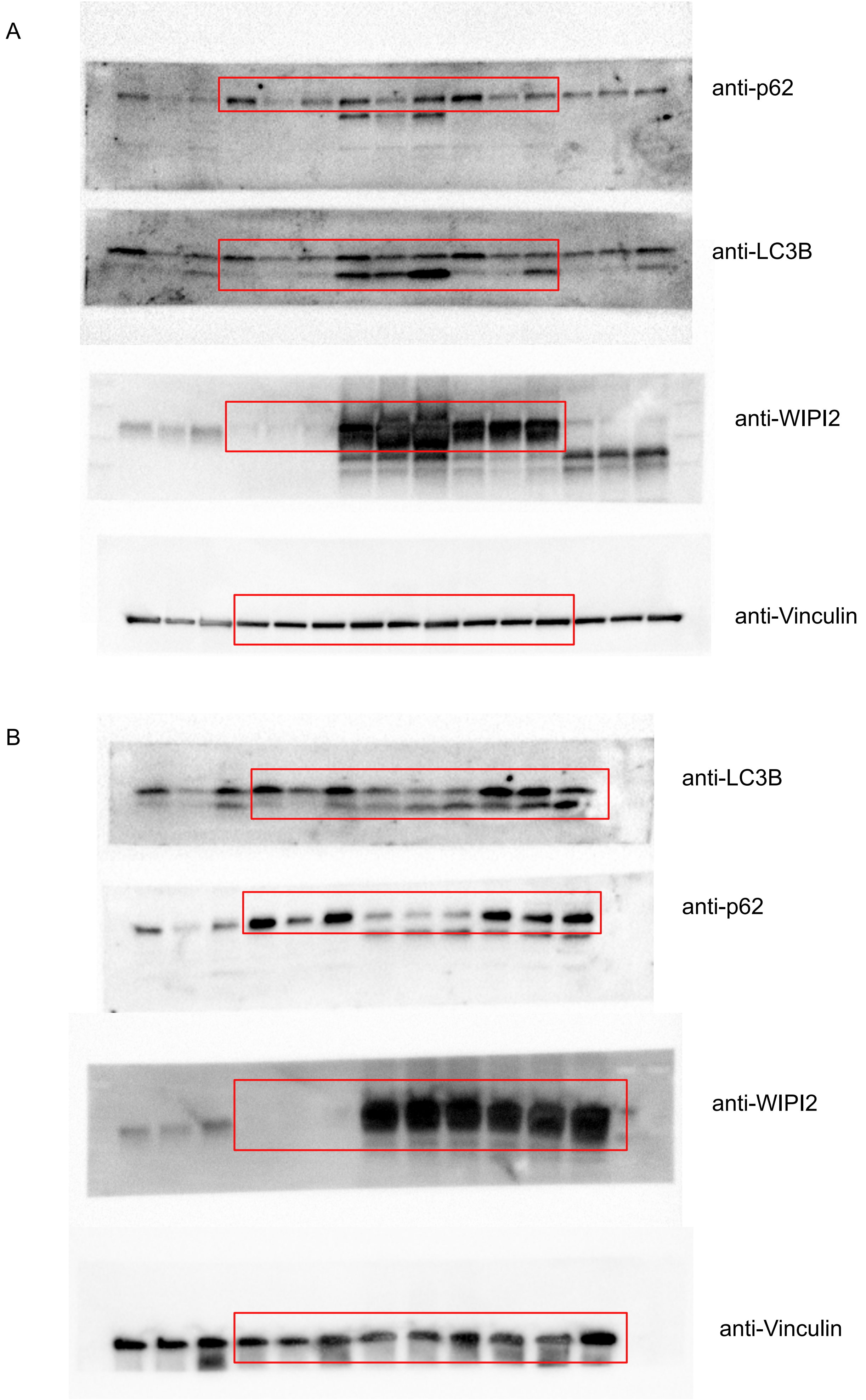

Supplementary figure 1. Un-cropped blots corresponding to (A) Figure 2A and (B) Figure 2B.
