# Supplementary material for: Homozygous missense WIPI2 variants cause a congenital disorder of autophagy with neurodevelopmental impairments of variable clinical severity and disease course
Source: Brain Commun. 2021 Sep 3;3(3):fcab183. doi: 10.1093/braincomms/fcab183 (PMC8453401; doi:10.1093/braincomms/fcab183)
Supplement: fcab183_Supplementary_Data [file fcab183_Supplementary_Data.zip › Supplementary Material.docx]

**Supplementary material for “Homozygous missense *WIPI2* variants cause a congenital disorder of autophagy with neurodevelopmental impairments of variable clinical severity and disease course”**

1. Supplementary Case Reports

2. Supplementary Tables

**1. Supplementary Case Reports**

**Family 1**

Family 1 presents with 3 affected children of Egyptian origin. Parents are healthy first cousins. Paternal and maternal ages were 40 and 37 years old, respectively. Apart from the reported patients, they had healthy male offspring. Pregnancy and delivery histories for all siblings were uneventful, with average growth parameters at birth and regular neonatal course.

Case F1-IV:1 was the oldest girl in the family, aged 13 years old at the first clinical evaluation. She had a severe psychomotor delay and mild hypotonia during infancy. At 2 years, the patient started to suffer from epilepsy with generalized myoclonic seizures. Additionally, tonic seizures with cyanosis started 2 years later. Multi-drug therapy with valproate, levetiracetam, clonazepam, and topiramate failed to control seizures. EEG showed bilateral temporo-parietal epileptogenic discharges with slow background activity. At 13 years the patient showed severe cognitive impairment, she could not vocalize or follow objects, and fairly responded to sounds. Her weight was 12.5 kg (-3.5 SDs), length 110 cm (-7 SDs) and head circumference 47 cm (-4.9 SDs). Dysmorphic features included a long face with a prominent chin, thick eyebrows, prominent nose, thick alveolar ridge, teeth deformities, and low set ears. In addition to the short stature, she had kyphoscoliosis, overlapping toes, contractures of hands and feet, and pes planus. Detailed neurological examination showed nystagmus, drooling, severe spasticity, brisk reflexes, and dyskinetic movements. Auditory brain stem evoked potentials were normal. The ophthalmologic evaluation showed a normal fundus. While visual evoked potentials showed delayed latency of central vision, her electroretinogram (ERG) was normal. Brain MRI performed at 7 years of age revealed corpus callosum hypoplasia (CCH), periventricular white matter signal anomalies, enlargement of the subarachnoid spaces and lateral ventricles, inferior cerebellar vermis hypoplasia, swelling with T2 hyperintensity of the cerebellar dentate nuclei, and platyspondyly of the cervical vertebrae (Fig. 1C). At age 10 years, the patient developed severe hemolytic anemia (with hemoglobin level as low as 6 mg%) requiring several blood transfusions and recurrent jaundice. Four years later, she was admitted to the pediatric intensive care unit due to a severe chest infection and died of respiratory failure. Karyotype and extended metabolic screening (including the dosage of organic acid in the urine) yielded normal results.

Case F1-IV:3 is the third-born in the family. He was 11.4 years old at the first clinical evaluation. The child met basic developmental milestones, being able to sit unassisted and crawl at the age of 1.5 years. Subsequently, a gradual regression was noted. Epilepsy started at the age of 3 years with myoclonic and tonic seizures, which recurred every week or less. Seizures were refractory to multidrug treatment with valproate, levetiracetam, and clonazepam. Clinical history also revealed a femur fracture at the age of 5 years. At 11.4 years, his weight was 11 kg (-3.1 SDs), length 106 cm (-5.8 SDs) and head circumference 46.5 cm (-4.9 SDs). He had facial dysmorphic features (including a long face with a prominent chin, thick eyebrows, prominent nose, thick alveolar ridge, teeth deformities, a double row of teeth, and large ears) and musculoskeletal abnormalities (kyphoscoliosis and long fingers and toes). Neurological examination revealed nystagmus, drooling, spasticity, brisk reflexes, dyskinesia, and hands and feet contractures. The patient appeared irritable and showed excessive crying. He did not show significant social interaction and could not follow objects, but a mild response to sounds was observed. EEG showed bitemporal epileptogenic discharges with slow background activity. Brain MRI performed at 3 years of age showed CCH with prevalent involvement of the posterior sections, mild reduced periventricular white matter, inferior cerebellar vermis hypoplasia, swelling with T2 hyperintensity of the dentate nuclei, and cervical platyspondyly (Fig. 1C). Extended metabolic investigations yielded normal results.

Case F1-IV:4 is the youngest female, aged 7.5-years-old at the time of the first clinical evaluation. She was diagnosed with a severe psychomotor delay since early infancy. She lacked head control, could not vocalize, and only occasionally showed social smiling after eye contact. Since infancy, she suffered from recurrent myoclonic seizures with temporo-parietal epileptogenic discharges and slow background activity on the EEG. Brain MRI performed at 6 years of age showed CCH with prevalent involvement of the posterior sections, mild reduced periventricular white matter, marked enlargement of the frontotemporal subarachnoid spaces, inferior cerebellar vermis hypoplasia, swelling with T2 hyperintensity of the dentate nuclei, and cervical platyspondyly (Fig. 1C). At 7.5 years, her weight was 11 kg (-3 SDs), length 101 cm (-4 SDs) and head circumference 47.5 cm (-3.1 SDs). She had mild dysmorphic facial features, such as a long face, prominent nose, long philtrum, retrognathia, abnormal teeth shape, thick alveolar ridge, and large ears with prominent antihelix. On neurological examination, nystagmus, spasticity, and brisk reflexes were observed. Metabolic investigations were normal.

**Family 2**

The proband (F2-IV:3) is a 5-year-old female born to consanguineous healthy parents. Family history was remarkable for a previous intrauterine fetal demise due to anencephaly. She was born at 30 weeks gestation after an uneventful twin pregnancy. Birth weight, length, and head circumference were normal. Her older sister and twin brother are both alive and healthy. At the age of 11 months, the patient presented with nystagmus and developmental delay. Electroretinography (ERG) and visual evoked potential (VEP) showed bilateral retinal and visual pathway involvement, leading to a possible diagnosis of cone-rod dystrophy. Brain MRI showed CCH with prevalent involvement of the posterior sections, hypoplasia of the anterior commissure, and mild reduced periventricular white matter with signal alterations. The inferior cerebellar vermis was small, and there was platyspondyly of the cervical vertebrae (Fig. 1C). Neurological examination revealed behavioral problems consistent with autism spectrum disorder (ASD) and visual impairment. She could sit independently at the age of 18 months and walk at the age of 2 years. She said her first words at the age of 3 years. Currently, she can walk if supported, with an ataxic gait. She has normal tone and reflexes. Her expressive and receptive language development is very limited. Behavioral problems include irritability, stereotyped movements, and self-injurious behavior. She was never diagnosed with seizures, and her EEG is normal. Physical examination at the age of 4.6 years did not show relevant dysmorphic features nor musculoskeletal abnormalities. Her growth parameters are normal. Metabolic work-up was unremarkable.

**2. Supplementary Tables**

**Supplementary Table 1. Phenotypic comparison associated with defects in *WIPI2*, *WIPI3* and *WIPI4* genes**

| **Gene** |  | ***WIPI2*** | ***WIPI3*** | ***WIPI4*** | |
| --- | --- | --- | --- | --- | --- |
| **Inheritance** |  | **AR** | **AR** | **X-linked** | |
|  | Disorder  (OMIM) | Intellectual developmental disorder with short stature and variable skeletal anomalies (IDSSA, #618453) | Neurodevelopmental disorder with spastic quadriplegia and brain abnormalities with or without seizures  (NEDSBAS, #617977) | Static encephalopathy of childhood with neurodegeneration in adulthood; (SENDA #300894) | |
|  |  | 6-8 cases | 10 cases | Stige et al., 2018  64 cases | Adang et al., 2020  123 cases |
| **Clinical findings** | DD | 6/6 (100%) | 7/10 (70%) | 64/64 (100%) | 74/123 (60.5%) |
|  | ID | 6/6 (100%) | 10/10 (100%) | 64/64 (100%) |  |
|  | Severity range | Mild to Profound | Severe to Profound | From asymptomatic carriers to profound | |
|  | Epilepsy | 3/6 (50%) | 5/10 (50%) | 42/62 (67.7%) | 32 (26.0%) |
|  | Movement disorders/spasticity/gait ataxia | Dystonia 3/6 (50%)  Gait ataxia 3/6 (50%) | Spastic quadriplegia – 6/10 (60%) | Dystonia 44/60 (73.3%)  Parkinsonism 35/58 (60.3%) | 1 (0.8%) |
|  | Microcephaly (primary or secondary) | Secondary  3/6 (50%) | Secondary 7/10 (70%) | 1/11 (9%) | 13/47 (27.7%) |
|  | Dysmorphic features | 5/6 (83%) |  | 11/16 (68.8%) | NA |
|  | Progressive cognitive decline | - | - | 44/59 (74.6%) | NA |
|  | Progressive loss of motor function | 1/6 (17%) | - | NA | 41/84 (51.2%) |
|  | Progressive loss of verbal function | - | - | NA | 27/71 (38.0%) |
|  | Disease course: static or progressive | Progressive and static | Progressive | Initially static with regression at the mean age of 27.2 ±5.7 | |
|  | Autistic features | 1/6 (17%) | - | NA | 1 (0.8%) |
|  | Rett-like features | - | - | 14/50 (28%) | NA |
| **Imaging findings** | Iron deposition | - | - | 55/61 (90.2%) | 82/159 (51.6%) |
|  | Cerebral atrophy | - | 5/5 (100%) | 44/63 (69.8%) | 104/159 (65.4%) |
|  | Cerebellar atrophy | ICV Hypoplasia  5/5 (100%) | - | 17/63 (27%) | 37/159 (23.3%) |
|  | Abnormal myelination | - | - | 6/63 (9.5%) | 45/159 (28.3%) |
|  | Reduced white matter volume | 5/5 (100%) | 5/5 (100%) |  |  |
|  | Abnormal corpus callosum | 5/5 (100%) | - | 2/63 (3.2%) | 60/159 (37.7%) |
|  | Dilated ventricles | 5/5 (100%) | 5/5 (100%) | 2/63 (3.2%) | NA |

Abbreviations: DD= developmental delay; ID= intellectual disability; ICV=inferior cerebellar vermis; NA= not available

**Supplementary Table 2. Facial dysmorphism and musculoskeletal findings in *WIPI2* patients.**

|  | **This study** | | | | **Jelani et al., 2019** | |
| --- | --- | --- | --- | --- | --- | --- |
|  | **F1-IV:1** | **F1-IV:3** | **F1-IV:4** | **F2-IV:3** | **NA** **(IV-4)** | **AA (V-3)** |
| **Facial dysmorphism** |  | | | | | |
| Rough brittle hair | - | - | - | - | + | N/A |
| Long face | + | + | + | - | - | - |
| Thick eyebrows | + | + | + | - | + | + |
| Large ears | - | + | + | - | N/A | N/A |
| Low-set ears | + | - | - | - | N/A | N/A |
| Prominent nose | + | + | + | - | + | + |
| Palate deformity | Thick alveolar ridge | Thick alveolar ridge | Thick alveolar ridge | - | - | - |
| Gingival hypertrophy | - | - | - | - | - | + |
| Teeth deformities | + | + (double row of teeth) | + | - | + | + |
| Enamel defect | + | + | + | - | + | + |
| Prominent chin | + | + | - | - | + | N/A |
| Progeroid features | - | - | - | - | + | + |
| **Musculoskeletal abnormalities** |  | | | | | |
| Kyphosis | **+** | **+** | **-** | - | **+** | **-** |
| Scoliosis | **+** | **-** | **-** | - | **-** | **-** |
| Clinodactyly | **+** | **+** | **+** | + | **+** | **+** |
| Long fingers | **-** | **+** | **-** | - | **-** | **-** |
| Thumb hypoplasia | **-** | **-** | **-** | - | **+** | **-** |
| Hand contractures | **+** | **+** | **+** | - | **-** | **-** |
| Pes planus | + | - | - | - | - | - |
| Long toes | - | + | - | - | - | - |
| Overlapping toes | + | + | - | - | + | - |
| Foot contractures | + | - | + | - | - | - |

Abbreviations: N/A = not available; Pt = patient.

**Supplementary Table 3. *In silico* analysis of known *WIPI2* variants.**

|  | ***WIPI2* variant**  **(NM_ 015610.4)** | **gnom**  **AD** | **dbSNP** | **Iranome** | **GME** | **In-house database†** | **GERP** | **CADD** | **SIFT** | **Polyphen2** | **Mutation Taster** | **Mutation Assessor** | **FATHMM-MKL** | **PROVEAN** | **REVEL** | **ACMG class**  **(criteria)** |
| --- | --- | --- | --- | --- | --- | --- | --- | --- | --- | --- | --- | --- | --- | --- | --- | --- |
| **Jelani et al., 2019** | c.745G>A; p.(Val249Met) | 0.000007213 (2 het) | rs756429763 | - | - | - | 5.57 | 25.8 | D  (0.001) | PD  (1) | DC  (1) | Medium (3.48) | D  (0.9942) | D  (-2.57) | P (0.6779) | V (PS3, PM1-2, PP1, PP3) |
| **This study**  **(Family I)** | c.551T>G; p.(Val184Gly) | - | - | - | - | - | 5.78 | 25.8 | D  (0) | PoD  (0.778) | DC  (1) | High  (3.58) | D  (0.9744) | D  (-6.57) | P  (0.693) | IV (PS4, PM2, PP1, PP3, PP4) |
| **This study**  **(Family II)** | c.724C>T  p.(Arg242Trp) | - | - | - | - | - | 5.67 | 24.8 | D  (0.005) | PoD  (0.566) | DC  (1) | High  (3.995) | D  (0.9819) | D  (-7.21) | P  (0.7429) | IV (PM1-2, PP3-4) |

Abbreviations: D = damaging; DC = disease causing; GME = Greater Middle East Variome Project; P = pathogenic; PD = probably damaging; PoD = possibly damaging; PS = pathogenic strong; PM = pathogenic moderate; PP = pathogenic supporting; NR=non reported. † In-house database of more than 10,000 exomes.
